# Grants to Medical Schools of London

**Published:** 1920-12-18

**Authors:** 


### Grants to Medical Schools of London.

In reply to a question in the House of Commons a short time ago, the Chancellor of the Exchequer furnished the following particulars of block grants to London hospital medical schools during the academic year 1919-20: Charing Cross, £1,000; King's College, £700; London, £6,000; Royal Free, £4,000; St. George's, £700; St. Bartholomew's, £5,000; St. Mary's, £1,900; St. Thomas's, £4,500; University College, £4,000; Westminster, £300; London School of Tropical Medicine, £1,100; Middlesex, £2,000; Royal Dental, £1,000. In addition, the following grants for clinical units were made: St. Bartholomew's (medical and surgical), £5,750; St. Thomas's (surgical), £2,330; and University College, £5,570. The staffs and salaries of these clinical units are: St. Bartholomew's, two directors, £2,000 each; two assistant directors, £750 each; two first assistants, £400 each; two second assistants, £350 each. St. Thomas's, director, £1,500; first assistant, £500; second assistant, £400; pathological assistant, £500. University College, surgical director, £2,000; two assistants, £650 each; medical director, £1,500; first assistant, £700; second assistant, £60.

As we go to press we learn that a general meeting of the Medical Officers of Schools Association is being held on Friday, December 17, at 4.30 p.m., at 2 Savoy Hill, Victoria Embankment, W.C. 2, to elect officers for 1921. Mr. R. C. Elmslie has been nominated for the office of President.

